# Unexpected air pollutants with potential human health hazards: Nitrification inhibitors, biocides, and persistent organic substances

**DOI:** 10.1016/j.scitotenv.2022.160643

**Published:** 2022-11-30

**Authors:** Johann G. Zaller, Maren Kruse-Plaß, Ulrich Schlechtriemen, Edith Gruber, Maria Peer, Imran Nadeem, Herbert Formayer, Hans-Peter Hutter, Lukas Landler

**Affiliations:** a University of Natural Resources and Life Sciences Vienna (BOKU), Department of Integrative Biology and Biodiversity Research, Institute of Zoology, 1180 Vienna, Austria; b TIEM Integrated Environmental Monitoring, 95615 Marktredwitz, Germany; c TIEM Integrated Environmental Monitoring, 44135 Dortmund, Germany; d University of Natural Resources and Life Sciences Vienna (BOKU), Department of Water, Atmosphere and Environment, Institute of Meteorology and Climatology, 1180 Vienna, Austria; e Department of Environmental Health, Center for Public Health, Medical University Vienna, 1090 Vienna, Austria

**Keywords:** Agrochemicals, Biocides, Off-target area, Pesticide drift, Exposure, Agriculture, Human toxicology, Air pollution, Toxic loads, Environmental risk assessment

## Abstract

To better understand the influence of land use and meteorological parameters on air pollutants, we deployed passive air samplers in 15 regions with different land use in eastern Austria. The samplers consisted of polyurethane PUF and polyester PEF filter matrices, which were analyzed for 566 substances by gas-chromatography/mass-spectrometry. In a previous article, we highlighted a widespread contamination of ambient air with pesticides that depends on the surrounding land use and meteorological parameters. Here we report that, in addition to agricultural pesticides, eight other substances were frequently detected in ambient air: Nitrapyrin, a nitrification inhibitor used to increase nitrogen use efficiency of fertilizers and banned in Austria since 1993; biocides against insects (DEET and transfluthrin) used mainly outside agriculture; piperonyl butoxide (PBO), a synergist mixed into pesticide formulations; and four industrially used polychlorinated biphenyls (PCBs), long banned worldwide. Concentrations of the detected substances were positively related to air temperature, but only slightly related to agricultural land use in the sampler’s vicinity. The city center showed the highest concentrations of biocides, PCBs and PBO, but also medium concentrations of nitrapyrin. Four sites had no air contamination with these substances; including two national parks dominated by grassland or forest, but also two sites with mixed land use. The potential human toxicity of the detected substances based on globally harmonized hazard classifications was high: seven substances had specific organ toxicity, six were cancerogenic, and two were acutely toxic; however, several substances had incomplete information of hazard profiles. Moreover, all substances were acutely and chronically toxic to aquatic life. We recommend that substances of different origins be included in the air pollution monitoring portfolio to comprehensively assess the potential hazards to humans and the environment.

## Graphical abstract

**Graphical Abstract:**
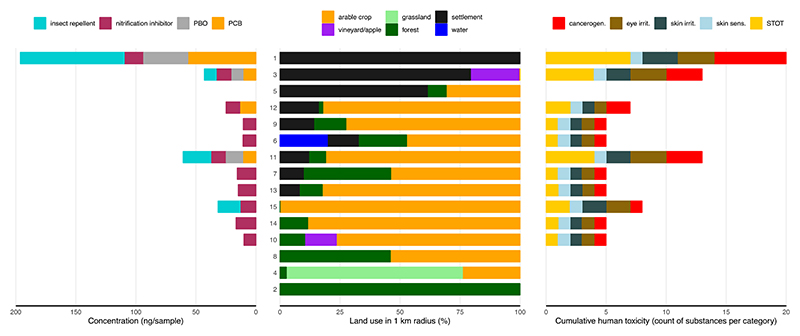

## 1 Introduction

Global chemical pollution has increased dramatically in recent decades, leading to various potential risks that exceed society's ability to conduct safety-related assessments and monitoring (Persson et al., 2022). When it comes to agriculture's contribution to air pollution, synthetic pesticides are often considered (Córdoba Gamboa et al., 2020; Dalvie et al., 2014; Kruse-Plaß et al., 2021; Veludo et al., 2022; Zaller et al., 2022). Pesticide drift can expose farm workers and bystanders (Paglia et al., 2021; Sapcanin et al., 2016) and is often found on non-agricultural sites (Cech et al., 2023; Linhart et al., 2019; Linhart et al., 2021) including in nature conservation areas (Brühl et al., 2021; Kruse-Plaß et al., 2021; Zaller et al., 2022).

In addition to pesticides, many other agrochemicals are introduced into the environment in agriculture, such as nitrification inhibitors. Nitrification inhibitors are used to increase the nitrogen-use efficiency of agricultural crops, as only about 50 % of the applied N fertilizer is absorbed by crops (Coskun et al., 2017). Moreover, many other chemical substances are also used as biocides in non-agricultural applications, such as insect repellents by private consumers or municipalities (Zaller, 2020). Finally, there are highly persistent organic pollutants that are commonly found in aquatic and terrestrial environments (Spurgeon et al., 2022). While biocides or persistent organic chemicals are considered as indoor air pollutants (Mull et al., 2015), very little is known about the contribution of these substances of various origins to outdoor air pollution.

In this study, we (i) assessed the environmental exposure to chemicals other than agricultural pesticides via passive air sampling, (ii) examined the influence of surrounding land use and meteorological parameters on local contamination levels, and (iii) reviewed the potential toxicological hazards of these exposures to humans and the environment based on globally harmonized hazard statements for the detected substances. We hypothesized that ambient air contamination with agrochemicals would be related to surrounding land use, as a result of application-related drift or via windblown soil particles (Mueller, 2015). In contrast, chemicals of non-agricultural origins should be less related to agricultural land use (Spurgeon et al., 2022) and more influenced by meteorological parameters (Bish et al., 2021; Linhart et al., 2019; Zaller et al., 2022). To our knowledge, the present study is one of the few that interactively examines multiple factors affecting exposure to non-pesticide chemicals and their potential risk to humans and the environment.

## 2 Materials and methods

### 2.1 Sampling locations

We selected 15 sampling sites in a cultural landscape in eastern Austria to represent a gradient from 0% to 100% agricultural use within a 1000 m radius. Agricultural land use included arable crops, managed grasslands, vineyards, or apple orchards. Additionally, one site was located in a city center 100 % surrounded by settlements, and two sites in National Parks of which one was surrounded by 100 % forest and the other one by 73 % unmanaged grassland. In order to protect the privacy of the landowners, the exact location of the selected sites cannot be given here.

Land use types were mapped within a 1 km radius of the sampler site based on publicly available CORINE Land Cover database (UBA Wien and EEA, 2018) and EUNIS Habitat Classification (Davies et al., 2004). To validate the most recent land cover database available from 2018 (while air sampling was conducted in 2020) we used orthophotos from 2020 with an accuracy of 29 cm to adjust for potential land use changes (Geoland, 2020). The following land use types were distinguished: arable crops, vineyards and apple orchards, grassland, forest, settlements, and water bodies. Mapping and analysis were performed using ArcGis 10.2.195, QGis 2.8.1, FRAGSTATS 4.296 and CHLOE201297.

We do not have data on the actual application of agrochemicals to agricultural fields during the study period. However, we assume that fields were managed according to so-called good agricultural practice (AMA,2006) which also included the application of agrochemicals. For grassland, forests, water bodies and settlements, we assume that pesticides were applied on spots only.

### 2.2 Passive air sampling

Passive air sampling was conducted in accordance with protocols of the Global Atmospheric Passive Sampling (GAPS) network (Schuster et al., 2021). We used passive air samplers consisting of two matrices per location: a polyurethane foam(PUF) and a polyester filter (PEF) sampler. Both matrices were placed at 1.8 m height. The mean distance between samplers was 99.3 ± 49.0 km (mean ± SD) with a minimum distance between samplers of 5.0 km and a maximum distance of 196.8 km. The PUF sampler consisted of a disk (diameter 14 cm, height 1 cm) under a reflecting chrome metal dome to avoid direct precipitation of dust and rain which affects the extent of collection of particle-associated chemicals (Markovic et al., 2015). The PEF sampler consisted of four disks (each diameter 8 cm, height 2 cm) placed under the dome and exposed to the air, also collecting particles. PUF disks were obtained from Tisch Environmental Inc. (Cleves, OH, USA), PEF disks from Freudenberg Filtration Technologies (Weinheim, Germany). The combined sampler consisting of PUF and PEF disks was built by TIEM Integrated Environmental Monitoring (Dortmund, Germany) and used to detect both volatile and particle-bound substances such as glyphosate (Kruse-Plaß et al.,2021;Zaller et al.,2022). Schematic illustrations and photographs of the sam-pler can be found in Kruse-Plaß et al. (2021).

Prior to sampling, PUF media were purified using acetone, petroleum ether and methanol (Shoeib et al., 2008). Non-exposed samples were analyzed for both PUF and PEF matrices to account for possible contamination.

Fifteen PUF and six PEF matrices were installed for up to eight months (March to November 2020), six PUF matrices were replaced approximately every two months during this period to account for seasonal variations in pesticide contamination (Supplementary Table S1). Matrices were changed by trained members of the research team using nitrile gloves and forceps according to clear instructions, stored in a cooler, and then placed in a freezer (−18 °C) until laboratory analysis.

### 2.3 Chemical analyses

Chemical analysis of the matrices was performed by the laboratory KWALIS (Fulda, Germany) registered with the German Accreditation Body (Deutsche Akkreditierungsstelle). Samples were analyzed for 566 chemical substances based on the active ingredients listed for plant-based foods in the official multi-method of the German Federal Office of Consumer Protection and Food Safety (BVL L 00.00–115: 2018–10; BVL (2018)). Chemical substances to be analyzed included pesticides, their metabolites, safeners, synergists, auxiliary materials, and compounds unrelated to pesticides that are known to exert adverse health effects and may be unintentionally present in agricultural products, such as polychlorinated biphenyls PCBs, or piperonyl butoxide PBO. The limits of detection were 10 ng sample^−1^ for all other non-pesticides reported in this study. A full list of target analytes including their detection limits can be found in Kruse-Plaß et al. (2021).

Following the protocol DIN EN 15662 (July 2018) chemical substances were analyzed by gas chromatography with mass spectrometry coupling (GC–MS) and/or liquid chromatography-tandem mass spectrometry (LC-MS/MS) in PEF after acetonitrile extraction/partitioning and purification with dispersive SPE sample preparation (QuEChERS) (BVL, 2018). Extraction of PUF was performed with dichloromethane in a Soxhlet extractor (Estellano et al., 2015; Yusà et al., 2009) and analyzed accordingly. Sampling results are reported in either ng sample^−1^ or calculated in concentrations per m^3^ of air, based on a default air sampling rate of PUF passive samplers of 4 m^3^ day^−1^ (Harner et al., 2014) with 257 days of sampler exposition in our study.

In the current study we focus only on non-pesticide substances, detailed results on pesticides and other substances analyzed are reported in Zaller et al. (2022).

### 2.4 Meteorological data

The meteorological data are based on the INCA dataset (Haiden et al.,2011) of the Austrian weather service ZAMG. INCA provides gridded mete-orological data with a temporal resolution of 1 h for the whole of Austria and a spatial resolution of 1 × 1 km. For the 15 locations, the representative INCA grid was selected and the mean was calculated for the vegetation period (10 March to 20 November) of 2020 for air temperature, windspeed, relative humidity, and solar radiation. For precipitation, totals were calculated.

### 2.5 Assessment of toxicity for humans and the environment

Potential human toxicological hazards linked to the detected substances were assessed based on the interpretation given in the Pesticide Properties Database (Lewis et al., 2016), the EU pesticide database (EC, 2022), PubChem(Kim et al., 2021), the International Agency for Research on Cancer (IARC, 2016), and the Office of Environmental Health Hazard Assessment of the Californian Environmental Protection Agency (OEHHA,2015). Categories distinguished were a substances' carcinogenicity, reproduction toxicity, endocrine disruption (EDC), acute toxicity, specific target organ toxicity STOT RE/SE (repeated/single exposure), skin irritation, skin sensitization, eye irritation. The interpretations of the majority of these databases are a summary of the main human health concerns across a number of issues using a ‘weight-of-the-evidence’ approach that emphasizes caution (Lewis et al., 2016).

Toxicity to the environment was based on acute and chronic aquatic toxicity (UN, 2021). Acute aquatic toxicity is assessed using a fish 96 h LC_50_ (OECD, 2019), a crustacea species 48 h EC_50_ (OECD, 2004) and/or an algal species 72 or 96 h EC_50_(OECD,2011). These species are considered as surrogate for all aquatic organisms and data on other species such as water lenses (Lemna spp.) may also be considered if the test methodology is suitable. Chronic toxicity data are less available than those for acute toxicity and the range of testing procedures less standardized. Data generated according to the OECD Test Guidelines for Fish Early Life Stage (OECD,2013),or *Daphnia* Reproduction(OECD,2012)and Algal Growth Inhibition (OECD, 2011) are considered.

### 2.6 Statistical analyses

All statistical analyses were performed in R (R Core Team, 2020). To reduce the risk of multicollinearity we first performed a principal component analysis (PCA, function in R: princomp) separately for the meteorological and land-use factors and used the principal components as independent variables in the statistical models. Because no non-pesticide substances were detected in PEF matrices we only used PUF data for statistical analyses.

We tested the effect of land-use and meteorological effects on substance concentrations (ng sample^−1^) using a model selection approach. The full model included the fixed dependent variables land-use and meteorological principal components (see above), specific substance and substance type as well as their interactions; in addition, the sampling location was added as a random factor. We used the buildglmmTMB function (buildmer package; Voeten (2020)) to select the model with the lowest Akaike information criterion and, within this framework, compared different distribution types (Tweedie (as implemented in glmmTMB), Gaussian and negative binomial). The Tweedie distribution performed best and was selected for the model. We checked model fits using QQ plots using the DHARMa package (Hartig, 2019). The ggpredict function in the ggeffects package (Lüdecke,2018) was used for model predictions and the function Anova.glmmTMB was used to build the ANOVA tables.

## 3 Results and discussion

In total, we found eight non-pesticide substances in air sampler matrix (only in PUF filters, none in PEF filters) with the highest measured concentration of DEET (diethyltoluamide, 47.66 ng sample^−1^) and the lowest concentration of nitrapyrin and PCB028 (each 10.00 ng sample^−1^) (Table 1). Sampling locations differed regarding the concentrations detected. Nitrapyrin was the most frequently found substance, DEET and PCB028 were found second most frequently; PCB052, PCB101, PCB153 and transfluthrin were found at only one site.

**Figure 1:**
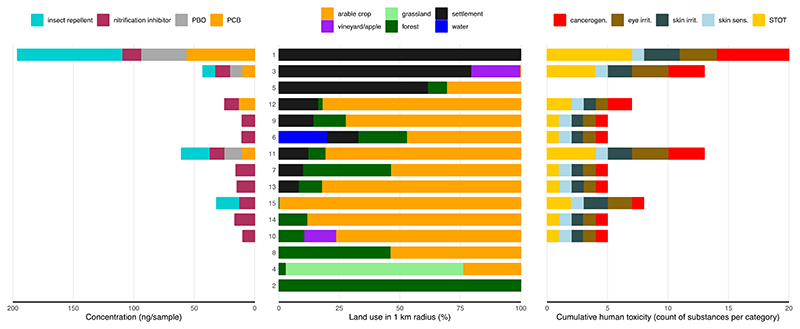
Concentration of substances other than agricultural pesticides (left) in passive air samplers (only PUF) located with different land use within a radius of 1000 m around the sampler (center) and cumulated human hazard scores according to various databases (right). Sites 2 and 4 were located in national parks, site 1 in a city center. The location index number was kept consistent with the previous publication (Zaller et al., 2022), however, the order is based on percentage of settlement.

Four sites had no contamination with these substances, including the two national parks (locations number 2 and 4; Fig. 1). The highest cumulative contamination was detected in the city center with a total of 196.29 ng sample^−1^ consisting of insect repellents, PCBs, PBO and nitrapyrin (Fig. 1). Supplementary Table S3 shows the sampling efficacy of the filter matrices used. The detection of only 8 substances out of the 566 target analytes can be explained by the rather high detection limit for these substances of 10 ng sample^−1^, since the sampling focus was on pesticides in ambient air (Zaller et al., 2022). Other studies using similar passive air sampling, but with lower detection limits, have detected a wide range of other non-pesticde contaminants (Jaward et al., 2004a, 2004b; Motelay-Massei et al., 2005; Pozo et al., 2016).

Statistical models showed that substance concentrations were influenced by the meteorological parameters radiation and temperature (Supplementary Fig. S1A, Supplementary Table S4). Less effective for substance concentrations was the land use in the surrounding area, e.g. proportion of forestry vs. arable land (Supplementary Fig. S1B, Supplementary Table S4).

All eight substances detected exhibited considerable acute and chronic aquatic toxicity (Supplementary Table S2).

Nitrapyrin (2-chloro-6-trichloromethyl pyridine), an organochlorine compound, is a bactericide for the soil-dwelling bacteria Nitrosomonas. Nitrapyrin is used as a nitrification inhibitor to improve the efficiency of nitrogen fertilizer or slurry and to reduce nitrate leaching and emissions of the climate-relevant gas N_2_O (Giacometti et al., 2020; Ruser and Schulz,2015; Subbarao et al., 2006; Wolt, 2000). Nitrapyrin has also been reported to increase resistance of maize plants to Fusarium and Aspergillus ear rot pathogens (Rácz et al., 2022). Nitrapyrin has also some phytotoxicity, resulting in reduced root production (Chambers et al., 1980). According to the European Chemicals Agency, between 100 and 1000 tons of nitrapyrin are applied annually in Europe, but the agency does not have data on the routes through which this substance is most likely to enter the environment (ECHA, 2022). Overall, nitrification inhibitors are applied to only about 1.6 % of arable land in the United States and only about 0.3% of arable land in Western Europe (Subbarao et al., 2006). The environmental fate of nitrapyrin after application is determined by sorption to soil and off-field transport via leaching and overland runoff (Woodward et al., 2019). Limited research has been conducted on the ecotoxicological effects of nitrogen inhibitors in general. Two commercial nitrogen inhibitors containing active ingredients other than nitrapyrin (e.g., 1H-1,2,4-triazole, 3-MP, or DMPP) showed inhibition of seed germination inhibition and root development in aquatic plants and impaired root development in several terrestrial plant species (Kösler et al., 2019). Nitrapyrin was also detected in two of 106 air samples from sites across Germany (at 12.8 and 14.6 ng sample^−1^) with passive air samplers similar to those used in the current study (Kruse-Plaß et al., 2021). Nitrapyrin has been banned in Austria since 1993 (RIS,1994), so the frequent detection is puzzling. Since nitrapyrin is highly volatile (Flessa et al., 2014), there is a possibility that it drifted into Austria from neighboring countries such as Hungary, which is only a few kilometers away from some study sites and where it is commonly used (Rácz et al., 2021). Of the two study sites closest to the Hungarian border, one had the highest concentration while the other was not contaminated, also indicating other influencing factors.

DEET and transfluthrin are active ingredients that are not approved for agricultural use in the study country, but are widely used biocides against insects, especially in non-agricultural settings. Consequently, DEET is generally among the most frequently detected compounds in the environment together with nanomaterials, pesticides, pharmaceuticals, industrial compounds, personal care products, and other chemicals (Stuart et al., 2012). Transfluthrin is a low persistent pyrethroid insecticide that is mainly used indoors against flies, mosquitoes, moths, and cockroaches (Khan et al.,2017). Laboratory studies with female rats show that a mixture of DEET and the pyrethroid permethrin promotes epigenetic transgenerational inheritance of pubertal abnormalities, testis disease, and ovarian disease in F3 generation animals (Manikkam et al., 2012). The highest concentrations of insect repellents in the city center suggest widespread residential or commercial use against irritating insects (Zaller, 2020).

Polychlorinated biphenyls (PCBs) have been used in hundreds of industrial and commercial applications. Because they are persistent organic pollutants, they were banned in Europe and elsewhere in the late 1970s (UNEP, 2019), but new production has recently been reported ´in North Korea (Lauby-Secretan et al., 2013). PCBs are ubiquitous in our environment and have reached the polar regions and even the sediments of the deepest region on Earth, the Mariana Trench, at 11,000 m water depths (Dasgupta et al., 2018). PCBs impair reproduction, immune function and long-term viability of orcas (Desforges et al., 2018) and have been detected in eggs of terrestrial and aquatic bird species (Provini and Galassi,1999). Occupational exposure used to be highest during the manufacture of PCBs; today, exposure can result from the demolition, dysfunction, or uncontrolled recycling of PCB-contaminated equipment (Lauby-Secretan et al., 2013). Worldwide monitoring programs have shown that PCBs are present in most human milk samples (Lauby-Secretan et al., 2013). Based on sufficient evidence of carcinogenicity in humans and experimental animals in terms of an increased risk of melanoma, non-Hodgkins lymphoma, and breast cancer, the International Agency for Research on Cancer classified PCBs as group 1 carcinogens to humans (IARC, 2016). Moreover, all PCBs can induce formation of reactive oxygen species, genotoxic effects, immune suppression, an inflammatory response, and endocrine effects to varying degrees and via different pathways (Lauby-Secretan et al., 2013). It is unclear to what extent air pollution with PCBs affects the health of wildlife and humans.

Piperonyl butoxide (PBO) is a synergist that enhances the efficacy of insecticides such as pyrethrins, pyrethroids, rotenone and some carbamates and has little insecticidal activity itself (Bernard and Philogène, 1993). In mammals and humans, it is toxic and is suspected of causing anorexia, carcinogenesis, convulsions, and skin irritation, as well as liver and kidney damage (PubChem, 2022). Even exposure to low doses have been shown to adversely affect aquatic organisms (e.g., blue crabs) by altering predator-prey interactions and increasing vulnerability to predation (Schroeder-Spain and Smee, 2019). The effects of PBO on terrestrial non-target organisms are little studied. In Germany, PBO was detected in 6 air samples among 106 study sites (Kruse-Plaß et al., 2021). As with other substances detected in this study, the role of PBO in air is difficult to assess, but in general, such chemical stressors may play an important but underappreciated role in affecting biodiversity in terrestrial and aquatic ecosystems (Brühl and Zaller, 2019).

In terms of the potential of concentration-independent hazards to human health, nitrapyrin was the most problematic of the eight substances detected in our samples, with statements for six hazard categories: Cancerogenicity, acute toxicity, specific target organ toxicity, skin irritation and sensitization, and eye irritation. DEET was the second most hazardous substance with four statements, PBO had three, PCBs had two, and transfluthrin had one human hazard statement (Supplementary Table S2). None of the detected substance had reproductive toxicity or endocrine activity statements. However, it should be noted that hazard profile information was incomplete for many substances, which was also noted for pesticides (Silva et al., 2022). Across all sampling locations, air in the city center had the highest cumulative human health hazard scores, followed by the site with the second highest percentage of settlements and a site with 81 % arable crops, 12 % settlement, and 7 % forest (Fig. 1).

Overall, we were surprised to find the banned nitrification inhibitor nitrapyrin so frequently in our air samplers. Unlike a previous study that looked at pesticides in the air (Zaller et al., 2022), the concentrations of the non-pesticide compounds in the current study were influenced mainly by meteorological parameters and only to a small extent by surrounding land use. Clearly, further research is needed to better understand how meteorological and application patterns interact. Our data show that air pollution is a mixture of chemicals originating from agriculture, residential consumers, and legacy sources of highly persistent industrial chemicals. We recognize that quantifying the hazard posed by these airborne substances is simplistic and difficult to extrapolate to realistic exposure scenarios. Although the concentrations detected were low, caution is warranted due to the health and environmental hazards associated with the detected chemicals and interactions with other global environmental changes under way such as air pollution, climate change, chemical pollution and biodiversity loss (Frumkin and Haines, 2019). To make our per-sample measurements more comparable to others, we converted concentrations per passive sampler to concentrations per m^3^ of air using a default air sampler rate of 4 m^3^ day^−1^ (Harner et al., 2014) for the duration of sampling (257 days). For nitrapyrin, this corresponds to an intake of 127 pg of a harmful substance by an adult breathing 10 m^3^ of air per day. Systematic monitoring of air and other media in conjunction with epidemiological assessments would be imperative to understand the effects of these substances on humans and the environment (Cech et al., 2023; Fritsch et al., 2022).

## Supplementary Material

Figure S1

Supplementary tables

## Figures and Tables

**Table 1:** Substances other than pesticides found in passive air sampler (only PUF matrix considered). Min values refer to lowest concentrations detected, when the substance was present. *N* = 15 sites were sampled. n.a…not applicable because only one sample. Calculated concentrations for an exposition of 257 days and a default air sampling rate for passive air sampler of 4 m^3^ day^−1^ (Harner et al., 2014).

Substance	Approved in agriculture in the study year	Found in number of samples	Concentrations				Rank based on mean conc.
			Min	Max	Measured mean ± SD (ng sample ^−1^)	Calculated mean ± SD (pg m^3^)	
DEET	Yes	4/15	10.0	47.7	25.2 ± 16.1	24.5 ± 15.7	1
PBO	No	3/15	10.7	37.0	20.7 ± 14.1	20.1 ± 13.7	2
PCB028	No	4/15	10.0	20.8	13.6 ± 5.0	13.2 ± 4.9	3
Nitrapyrin	No	11/15	10.0	16.6	13.0 ± 2.4	12.6 ± 2.3	4
PCB052	No	1/15	11.0	n.a.	n.a.		8
PCB101	No	1/15	13.2	n.a.	n.a.		6
PCB153	No	1/15	11.7	n.a.	n.a.		7
Transfluthrin	No	1/15	38.9	n.a.	n.a.		5

## Data Availability

Data will be made available on request.

## Highlights

- Passive air sampling detected 8 nonpesticide chemicals along a land use gradient.
- Substances found: a nitrogen inhibitor, biocides, and persistent organic chemicals.
- Concentrations increased with air temperature; influence of land use was small.
- Highest concentrations in a city center; no contamination in two national parks.
- Potential hazards for humans and aquatic life exist from airborne deposition.

## Availability of data and materials

The datasets used and/or analyzed during the current study are available from the corresponding author on reasonable request.
