# Supplementary figures and images for: Unexpected air pollutants with potential human health hazards: Nitrification inhibitors, biocides, and persistent organic substances
Source: Sci Total Environ. Author manuscript; Available in PMC 2023 Apr 7. (PMC7614393; doi:10.1016/j.scitotenv.2022.160643)

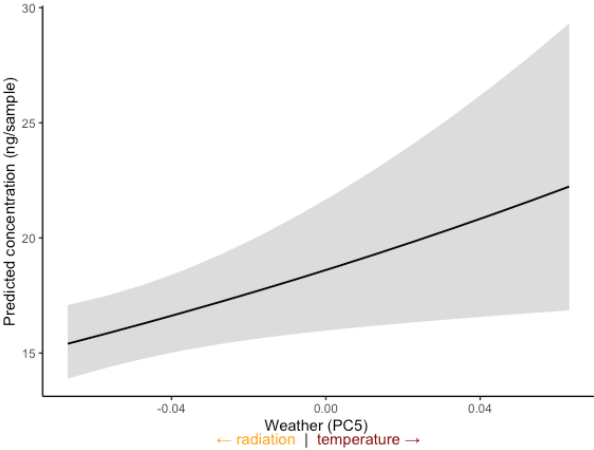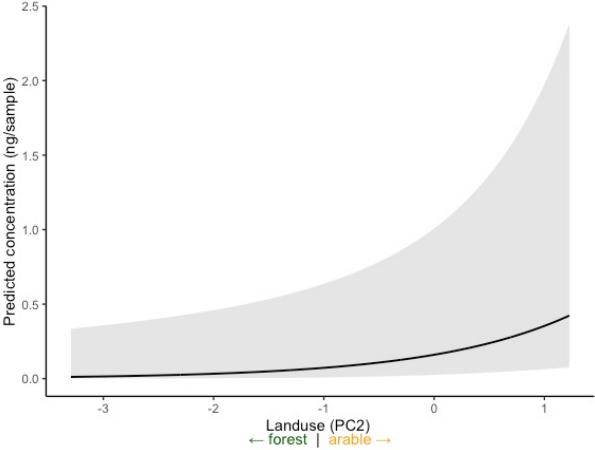

Supplement: Figure S1 [file EMS172741-supplement-Figure_S1.pdf]
