# Supplementary material for: Unexpected air pollutants with potential human health hazards: Nitrification inhibitors, biocides, and persistent organic substances
Source: Sci Total Environ. Author manuscript; Available in PMC 2023 Apr 7. (PMC7614393; doi:10.1016/j.scitotenv.2022.160643)
Supplement: Supplementary tables [file EMS172741-supplement-Supplementary_tables.docx]

**Supplementary Tables S1-S4 – Zaller et al. Unexpected air pollutants…**

**Table S1.** Overview of exposition times of the two air sampler matrices polyurethan foam (PUF) and polyethylene foam (PEF).

| Sample | Number  of sites | Start exposition  year 2020 | End exposition  year 2020 | Duration of exposition  (mean days) |
| --- | --- | --- | --- | --- |
| Polyurethan foam (PUF) | 15 | 02-13 Mar | 17-21 Nov | 257 |
| Polyethylen foam (PEF) | 6 | 07-10 Mar | 18-20 Nov | 256 |

**Table S2 Human health characteristics of non-pesticide substances collected with passive samples.** Classifications according to the Global Harmonized System (UN, 2021) within the PubChem database <https://pubchem.ncbi.nlm.nih.gov/ghs/>. n.i. means no information was available.

| Substance | Cancerogen. | Acute toxic | STOT RE/SE | Skin  irrit. | Skin sens. | Eye irrit. | Aquatic Acute | Aquatic Chronic |
| --- | --- | --- | --- | --- | --- | --- | --- | --- |
| Deet | n.i. | 4^1,2,3)^ | 3^4)^ | 2^1,2,3,4)^ | n.i. | 2^1,3)^/2A^2)^/2B^4)^ | 3^4)^ | 3^2)^ |
| Nitrapyrin | 2^3)^ | 3^4)^/4^1,3,4)^ | 2,3^4)^ | 2^4)^ | 1^3)^ | 2A^3,4)^/2B^4)^ | 1^2)^/2^4)^/ 2^4)^3^3)^ | 1^2)^/2^1,2,4)^/ 2^1,4)^3^3)^ |
| PBO | 2^4)^ | n.i. | 2/3^4)^ | n.i. | n.i. | 2B^4)^ | 1^4)^ | 1^4)^ |
| PCB028 | canc.^*)^ | n.i. | 2RE^2)^ | n.i. | n.i. | n.i. | 1^2)^ | 1^2)^ |
| PCB052 | canc.^*)^ | n.i. | 2RE^2)^ | n.i. | n.i. | n.i. | 1^2)^ | 1^2)^ |
| PCB101 | canc.^*)^ | n.i. | 2RE^2)^ | n.i. | n.i. | n.i. | 1^2)^ | 1^2)^ |
| PCB153 | canc.^*)^ | n.i. | 2RE^2)^ | n.i. | n.i. | n.i. | 1^2)^ | 1^2)^ |
| Transfluthrin | n.i. | n.i. | n.i. | 2^1,2,3)^ | n.i. | n.i. | 1^1,2,3)^ | 1^1,2,3)^ |

Sources cited in PubChem: 1) EC (2022b), 2) ECHA (2022a), 3) HCIS (2022), 4) NITE (2022), *) IARC (2016).

**Table S3 Overview of number of non-pesticides detected with passive air sampler using polyurethan foam (PUF) and polyethylene foam (PEF) matrices.**

| Parameter/ | Sampling method | | |
| --- | --- | --- | --- |
| Number of… | PUF | PEF |  |
| Samples | 15 | 6 |  |
| Non-agricultural substances detected | 8 | 0 |  |
| Detected substances per site (median) | 0 – 8 (1) | 0 |  |

**Table S4. Statistical analysis of total numbers and concentrations of pesticides in passive air samplers (PUF only) in response to land use in the surroundings and meteorological parameters. Significant effects in bold.**

| Predictors for concentrations detected | Chisq | df | p |
| --- | --- | --- | --- |
| Precipitation <> Temperature | 13.21 | 1 | **<0.001** |
| Substance type | 11.97 | 3 | **0.0075** |
| Forest <> arable land | 5.30 | **1** | **0.00213** |
| Precipitation <> Temperature by Substance type | 27.55 | **3** | **<0.001** |

<> denotes the main contributors to the included principal component axis, i.e. precipitation <> temperature together with a significant p-value means that concentrations were significantly influenced by an axis from high precipitation and low temperature towards low precipitation and high temperature.

**References**

HCIS, 2022. Safe work Australia. Hazardous Chemical Information System (HCIS). http://hcis.safeworkaustralia.gov.au/HazardousChemical. (Accessed 25 September 2022).

NITE, 2022. National Institute of Technology and Evaluation Japan. GHS classification results. https://www.nite.go.jp/chem/english/ghs/ghs_ver201410.html. (Accessed 25 September 2022).
